# Indications and Clinical Outcomes of Transoral Robotic Surgery and Free Flap Reconstruction

**DOI:** 10.3390/cancers13112831

**Published:** 2021-06-06

**Authors:** Philippe Gorphe, Stéphane Temam, Antoine Moya-Plana, Nicolas Leymarie, Frédéric Kolb, Apolline Bout-Roumazeilles, Quentin Qassemyar, Nadia Benmoussa, Jean-François Honart

**Affiliations:** 1 Department of Head and Neck Oncology, Gustave Roussy Institute, University Paris-Saclay, 94800 Villejuif, France; stephane.temam@gustaveroussy.fr (S.T.); antoine.moya-plana@gustaveroussy.fr (A.M.-P.); nadia.benmoussa-rebibo@gustaveroussy.fr (N.B.); 2 Department of Plastic and Reconstructive Surgery, Gustave Roussy Institute, University Paris-Saclay, 94800 Villejuif, France; Nicolas.leymarie@gustaveroussy.fr (N.L.); Apolline.bout-roumazeilles@gustaveroussy.fr (A.B.-R.); jeanfrancois.honart@gustaveroussy.fr (J.-F.H.); 3 Plastic and Reconstructive Surgery, UC San Diego, University of California, La Jolla, CA 92121, USA; frkolb@health.ucsd.edu; 4 Department of Plastic Surgery, Tenon Hospital, AP-HP, 75020 Paris, France; Quentin.qassemyar@aphp.fr

**Keywords:** oropharyngeal neoplasms, robotic surgery procedures, free tissues flaps, reconstructive surgical procedures, postoperative complications, quality of life

## Abstract

**Simple Summary:**

Transoral robotic surgery (TORS) with spontaneous healing is associated with improved quality of life as compared to traditional open surgery in small pharyngeal tumors. Improved surgeon experience allows very large or very complex resections, such as in a previously irradiated field where spontaneous healing is functionally insufficient or is at high risk of postoperative complications. We demonstrated very satisfactory feasibility and postoperative outcomes with a free flap microvascular reconstruction in this category of patients. TORS and free flap reconstruction has a place as a standard of care in a number of complex situations.

**Abstract:**

We reviewed the indications, peroperative feasibility, and postoperative clinical outcomes of our first 50 consecutive patients who underwent free flap reconstruction after TORS for complex pharyngeal defects at our institution. We analyzed indications according to previous radiotherapy, the size of the resection, and the transoral exposure of critical structures. We reviewed surgical data, postoperative complications, and functional outcomes comprising tracheostomy and alimentation management. Indications were upfront surgery (34%), a second primary surgery after radiotherapy (28%), or salvage surgery after chemoradiotherapy failure (38%). Localizations were the tongue base (44%), tonsillar fossa (28%), pharyngeal wall (22%), and soft palate (6%). T-classifications were T1 (6%), T2 (52%), T3 (20%), and T4 (22%). The mean length of the surgery was 574 min. Two patients were intraoperatively converted to a conventional approach at the beginning of the learning curve. In conclusion, TORS and free flap reconstruction in complex situations were associated with low rates of postoperative complications and satisfactory functional outcomes. They were, however, associated with a renewed learning curve.

## 1. Introduction

Since the worldwide adoption of transoral robotic surgery (TORS) using the da Vinci^®^ Surgical System after its Food and Drug Administration (FDA) approval for the treatment of T1–2 laryngeal and pharyngeal tumors in 2009, there has been an increase in surgery for small oropharyngeal cancers compared to non-surgical treatments in situations where it could be expected to improve outcomes [1,2]. Compared to an open approach, TORS has been shown to result in fewer postoperative complications and fewer adverse effects on quality of life associated with a transoral approach and healing by secondary intention [3]. This is thought to be due to the advantages of assistance by a robotic device with a 3D ‘in-field’ endoscope and EndoWrist^®^ articulated instruments compared to ‘out-of-the-field’ conventional surgery [4,5,6,7,8,9,10,11,12,13]. In oropharyngeal carcinomas, TORS with or without adjuvant has been used mainly for the surgical management of limited-size primary tumors on tongue bases or on tonsils with variable rates of early-stage and advanced-stage disease depending on the metastatic lymph nodes, resulting in very good functional and oncological outcomes [14]. In recent years, as a result of technological improvements and the increase in surgical experience with TORS, a number of teams have extended the size and complexity of resections [15,16,17]. However, TORS has not been developed to reduce the resection itself, and there is no rationale to expect smaller tissue defects according to the transoral surgical approach or the use of a robotic device. Potential postoperative complications and adverse effects on quality of life associated with the surgical defect remain issues that need to be addressed in complex surgical procedures, such as large resections, the exposure of critical structures, or salvage surgery after radiotherapy. The reconstruction of the pharyngeal defect with methods that range from a local flap to a distant free flap have been proposed by a small number of teams with published evidence of feasibility in TORS, and a consensual algorithm and classification system for transoral oropharyngeal defects was proposed by de Almeida et al. as a way to assist with the reconstruction decision-making process according to the local and general conditions [18,19,20]. At our institution, we started using TORS and free flap reconstruction based on our experience with reconstruction in conventional head and neck surgery in situations where it was expected to result in improved outcomes in terms of postoperative complications or quality of life. Our aim in this study was to review the first 50 cases that we performed involving TORS and free flap reconstruction and describe their indications and clinical outcomes after surgery.

## 2. Materials and Methods

This study was undertaken in accordance with the World Medical Association—Declaration of Helsinki—ethical principles for medical research, after having received approval from the local Research Ethics Committee. This was a retrospective observational cohort analysis. The patients analyzed in this study were operated on using the da Vinci^®^ Xi surgical system (Intuitive Surgical, Sunnyvale, CA, USA). From the first patient operated on until the CE marking of the da Vinci^®^ Xi for transoral surgery in July 2017, all of the patients were included in the prospective registered trial TORS-Xi NCT02517125, N°ID-RCB 2015-A00173-46. After July 2017, the patients were enrolled in the TORS-Xi trial based on whether they agreed to be enrolled. All the patients signed an informed consent form. We conducted an intention-to-treat analysis in the current study, meaning that we also included patients for whom surgery was converted intraoperatively from a transoral to an open approach at the beginning of our learning curve. 

Indications for TORS and free flap reconstruction used a defect-based algorithm that we developed, which was based on previous radiotherapy, the size of the resection in terms of oropharyngeal sub-units, and the medical conditions of the patient. Schematically, oropharynx defects are classified as type I when centered on the tonsillar fossa, type II when centered on the tongue base, and type III when centered on the posterior wall. Type I subunits are: (IA) resection limited to the tonsillar fossa, (+B) extended to the glossotonsillar sulcus and the tongue base, (+C) extended to the cheek, (+D) extended to the soft palate and the uvula, (+E) extended to the lateral and posterior wall, and (+deep) extended to the parapharyngeal space. Type II subunits are: (IIA) resection limited to the ipsilateral tongue base, (+B) extended to the glossotonsillar sulcus and the tonsillar fossa, (+C) extended to the contralateral tongue base, (+D) extended to the posterior mobile tongue and the posterior floor of the mouth, (+E) extended to the supraglotic larynx, and (+deep) extended to the comprehensive ispilateral tongue base muscles until the exposure of the complete ipsilateral hyoid bone and the pre-epiglottic space. Type III subunits are: (IIIA) the resection of the central posterior wall from one posterior pillar to the other, (+B) extended to the upper limit of the oropharyngeal posterior wall, and (+C) extended to the lower limit of the oropharyngeal posterior wall. In fit patients with no previous radiotherapy, a free flap reconstruction is generally recommended when at least 3 subunits are resected or at least 2 posterior wall subunits. In fit patients who have previously received radiotherapy, a free flap is preferred when at least 2 subunits are resected and is discussed when only 1 subunit is resected in comparison with a local FAMM flap. In patients unfit for free flaps, an alternative method of reconstruction is preferred.

The descriptive analysis characterized the studied population in terms of frequencies, percentages, medians, and ranges; we did not perform inferential statistics, which would have been irrelevant due to the small sizes of the subgroups. We did not analyze the oncological or survival outcomes, given the large range of settings from primary surgery to salvage after chemoradiotherapy failure and given the diversity of the histopathologies. Quality of life and functional outcomes were detailed regarding the evolution of airway and feeding management.

## 3. Results

### 3.1. Patients and Indications

The first 50 patients who underwent TORS and free flap reconstruction at our institution were operated on between 16 September 2015 and 22 April 2020. Their characteristics are reported in Table 1. Seven patients had a synchronous cancer: one lung adenocarcinoma that was operated prior to the TORS, one colon adenocarcinoma that was operated on after the TORS, one esophageal carcinoma that was irradiated after the TORS, one supraglottic carcinoma that was operated on at the same time as the TORS, one glottic carcinoma that was operated on with an endoscopic CO_2_ laser before the TORS, one laryngeal carcinoma that was irradiated after the TORS, and one sinus pyriform carcinoma that was operated on with an endoscopic CO_2_ laser before the TORS. Thirty-three patients (66%) underwent surgery after a previous head and neck radiotherapy, in a mean time of 5.1 years (range, 4 months–19 years). Four patients already had a chronic tracheostomy as a previous sequela, of whom one patient was laryngectomized. The remaining 46 patients were tracheotomized at the time of the TORS. 

### 3.2. Peroperative Feasibility

All of the TORS resections were performed by the same senior head and neck surgeon (PG). The free flap reconstructions were performed by five senior reconstructive surgeons. Two patients underwent an intraoperative conversion to a lip-split mandibulotomy approach, by the head and neck surgeon for one patient and by the reconstructive surgeon for the other patient. The first patient converted was our third TORS patient with a free flap reconstruction. This patient was a 71-year-old man with no previous radiotherapy who had a T4aN1M0 p16-negative squamous cell carcinoma of the tongue base that extended to the glossotonsillar sulcus. The difficult exposure of the massive submucosal tumor infiltration for a satisfactory resection meant that an open conversion for conventional surgery was preferable. After this patient, we developed a refined procedure, referred to as a cervico-transoral robotic oropharyngectomy, to overcome the limitations of the absence of haptic feedback with the robotic device for a complete en-bloc transoral resection of deep spaces along with the specimen while optimizing vascular safety [21]. The second patient converted was our 19th patient, a 72-year-old patient with no previous radiotherapy who had a T4aN2bM0 p16-negative SCC of the tongue base that extended to the glossotonsillar sulcus. The resection was successfully performed with a cervical-transoral robotic approach. However, the difficult intraoral flap inset and conformation meant that an open conversion for reconstruction was preferable. Notably, this was the second TORS and free flap reconstruction for this senior reconstructive surgeon, and we refined our team’s procedure for tongue base reconstruction. After the tongue base specimen resection, the head and neck surgeon introduced three long Vicryl^®^ 2-0 sutures on the lower surgical field borders using the robotic device: one suture at the posteromedial edge, usually at the angle between the contralateral tongue base and the glossoepiglottic sulcus; one suture at the lateral edge, usually at the lower glossotonsillar sulcus or at the three-fold region; and one suture between the two of them. The sutures were used by the reconstructive surgeon to progressively insert the flap transorally until it contacted the deeper tongue base section. In all of the patients, we performed an in-out flap inset: the flap was first inserted transorally, and the pedicle was passed in-out in the neck. The flap was then held in place by a small number of stitches, and microvascular sutures were performed. In tongue base surgery, as described above, the first stitches were placed at the lower borders of the flap; by contrast, in tonsillar fossa surgery and posterior pharyngeal wall surgery, the first 3-0 Vicryl^®^ stitches were placed at the upper border. The flap pedicle was channeled from the inside to the outside by a pharyngotomy, and conventional vascular microsutures could be performed on the external carotid artery system and the internal jugular vein system. After this, flap conformation and suturing could readily be performed transorally with robotic assistance whenever needed. 

**Figure 1:**
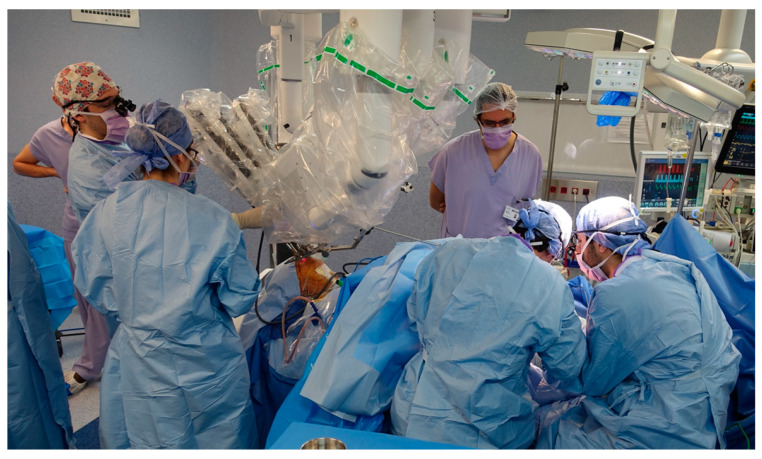
Situational image of the surgical room for two-team concurrent work in transoral robotic surgery and free flap reconstruction: the head and neck surgeon has performed a neck approach for vascular dissection and is installing the da Vinci^®^ Xi surgical system for transoral resection of the tongue base tumor while at the same time the reconstructive surgeons are harvesting a thin anterolateral thigh flap.

The flaps that were used are reported in Table 2. All of the procedures could be performed using a two-team approach due to the limited footprint of the da Vinci^®^ Xi surgical system in TORS (Figure 1) [22]. 

The median size of the defect measured transorally was very much higher than the mean size of the tumor at pathological examination (Table 2). This was due to the immediate lateral retraction of remaining tissues after the completion of the resection that we learned to take into account when planning the size of the flap skin paddle. The robotic device was used for flap suturing in 36 (72%) of the 50 patients analyzed in this series. The anatomical structures for which it proved to be useful were the structures below the upper border of the suprahyoid epiglottis: the lower posterior pharyngeal wall, the glossoepiglottic sulcus, the lateral pharyngoepiglottic fold, the three-fold region, the lateral pharyngeal wall at the oro-hypopharyngeal junction between, and the lower glossotonsillar sulcus. The mean duration of the surgery was 574 min (range, 29–820). The surgical defect comprised a mean of 2.98 anatomical subunits of the oropharynx. 

### 3.3. Postoperative Clinical Outcomes

During the hospitalization, nine patients (18%) had to be reoperated for a local complication. Two patients were reoperated for a hemorrhage; three patients (6%) had sutures for a flap disunion at the anterior or at the upper edge; and four patients (8%) had a free flap failure—two anterolateral thigh flaps, one radial forearm flap, and one medial sural artery perforator flap, of whom one had an immediate second free flap and three had a pectoralis major flap. No fistula occurred in patients in this series. Eleven (22%) patients had a postoperative pulmonary infection that required antibiotics. Two patients exhibited delirium tremens. Two patients decompensated an atrial fibrillation, one patient had a stroke, and two patients died from multivisceral failures due to severe pulmonary infections, both operated for salvage surgery after chemoradiation failure. The main categories of complications are reported in Table 3, stratified on the setting of the surgery.

**Figure 2:**
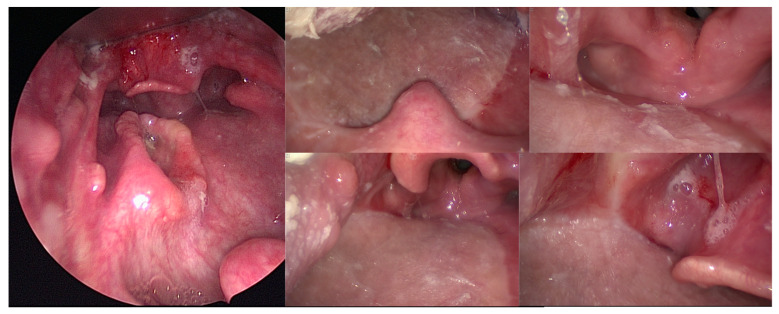
Endoscopic views of a 70-year-old patient with a 39 mm T2N0 p16-negative oropharyngeal carcinoma of the posterior pharyngeal wall who underwent transoral robotic surgery for resection of the posterior and lateral pharyngeal wall, bilateral neck dissection, reconstruction with a thin anterolateral thigh free flap, and postoperative radiotherapy. Forty-four months after the surgery, he was still alive and disease-free, with a normal oral diet and a very satisfactory quality of life.

**Figure 3:**
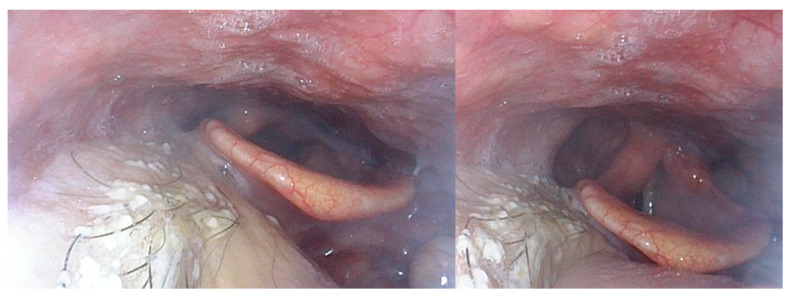
Nasofibroscopic views of a 60-year-old patient with a T3N0 hyalinizing clear-cell carcinoma of the tongue base. He underwent transoral robotic surgery and reconstruction with an anterolateral thigh free flap and did not require postoperative radiotherapy. Fourteen months after the surgery, he had a normal oral diet and speech and a very satisfactory quality of life.

Of the 46 patients who were tracheotomized at the time of surgery, excluding the two patients who died postoperatively, 100% were successfully decannulated in a median time of 12.5 days (range, 4–372), although the patient who had a postoperative stroke and a patient with chronic pulmonary aspergillosis needed a further recannulation for recurrent aspiration and for chronic respiratory insufficiency, respectively. The median time before being able to start at least partial oral alimentation was 17 days (range, 8–840). All of the patients began swallowing rehabilitation with a speech therapist during their hospitalization, and they continued with outpatient re-education. The mean length of hospitalization was 25 days (range, 10–52). All of the patients who were deemed to be candidates for postoperative radiotherapy (*n* = 15) or reirradiation (*n* = 11) were able to undergo the required treatment within six weeks postoperatively. The median follow-up time was 15 months. The rate of at least partial persistent enteral feeding at the last follow-up was 30.2% and this was higher with salvage surgery and surgery for a second primary cancer (35.3% and 33.3%, respectively) than with surgery for a first localization (23.5%). Posterior pharyngeal wall localizations were at high risk of persistent enteral tube feeding (62.5%) compared to tongue base (35%), tonsillar fossa (8.3%), and soft palate (0%) localizations. Highly critical key elements for efficient oral alimentation were the restoration of the tongue base volume, the preservation of laryngeal ascension at swallowing in case of a history of radiotherapy, the lateral unfolding of the sinus piriform at swallowing due to the preservation of the topography of the lateral pharyngoepiglottic fold, and the preservation of the sensitivity of the junction between the oropharyngeal and the hypopharyngeal posterior wall (Figure 2 and Figure 3).

## 4. Discussion

The goals of reconstruction in head and neck surgical oncological surgery are twofold. Firstly, the objectives are to improve quality of life. This comprises functional results such as swallowing, breathing, speech, oral competency, mastication, avoiding aspiration, acceptable aesthetic results, and a continuation of social life [23]. Secondly, the goals of reconstruction are to reduce treatment complications. These comprise postoperative complications such as bleedings, healing problems, fistulas, and infections, as well as oncological complications such as the risk of delaying postoperative treatments and the risk of death. The decision for reconstruction is usually based on the size of the defect, the exposure of critical anatomical structures such as the internal carotid artery, a previous history of head and neck radiotherapy, and the medical conditions of the patient. These collectively serve as a key for deciding on the reconstruction method used in daily practice, notably between a pedicled flap and a free flap in large soft-tissue defects. The feasibility of the use of free flaps after TORS has been demonstrated in a small number of case series, with reports of favorable outcomes [18,24,25,26,27,28,29]. Therefore, based on the previously published evidence-based risk of complications in TORS, the available experience-based reconstruction algorithms in TORS, and our experience in reconstructive surgery with approximately two hundred free flaps for head and neck reconstruction per year, we introduced TORS and free flap reconstruction at our institution in a defect-based decision-making process that was rationally based on the same goals and decision criteria as those used for conventional surgery [10,18,30,31,32,33]. The thorough analysis of the pitfalls that we encountered helped us to improve our standards. The two patients who were intraoperatively converted to a conventional approach would presently be able to undergo a successful cervical-transoral robotic pharyngectomy and free flap reconstruction, which would improve their quality of life. In a matched comparison of 18 patients who underwent TORS and free flap reconstruction versus patients who underwent a lip-split mandibulotomy approach, Biron et al. did not find a significantly decreased rate of postoperative complications or of tracheostomy decannulation time [28]. However, their reported rates of complications were very low, with only one patient experiencing a hematoma. This may be explained by the 88.9% and 93.1% rates of p16-positive patients in TORS and mandibulotomy groups, respectively. This constitutes populations with lower smoking and lower severe comorbidities than traditional p16-negative head and neck patients. By comparison, two thirds of the patients in our study population had received previous radiotherapy, resulting in known increased risks of complications. However, as in the series from Biron et al., no patients experienced postoperative fistula and the rate of chronic tracheotomy was low, which was very satisfactory for the quality of life of patients in this setting. In the largest series of patients undergoing TORS and free flap reconstruction to date, Hatten et al. reported the outcomes of 42 patients operated on [34]. Their patients cohort comprised 9.5% of patients previously treated with radiation therapy for a head and neck cancer. The mean flap size was 55.9 cm^2^ (compared to 56 cm^2^ in our series), the decannulation rate was 95%, and the functional outcomes they reported were very similar to ours. They experienced postoperative fistulas in 7.1% of patients. However, they reported large enough pharyngotomies following resection to allow reconstruction without the need for robotic instrumentation. We agree with the requirement of sufficiently wide resections for optimizing deep margins, especially in infiltrating tongue base tumors, and these differences between series may be due to the deviation of the patients’ characteristics rather than the surgical techniques used.

Our study has limitations that must be pointed out. This was a single-center retrospective cohort that was non-controlled and that had inclusion biases such as the high rate of patients who had previously undergone radiotherapy. We did not investigate the extra cost of the procedure related to use of the robotic system, although it may be offset by the benefits of reduced postoperative complications. Moreover, it is possible that these benefits also translate into oncological advantages, given the feasibility of postoperative radiotherapy in all of the patients compared to delays caused by local healing difficulties with open approaches with or without mandibulotomy [35]. We did not analyze the oncological outcomes due to the diversity of histopathologies in our series and the high rate of salvage surgery. Further studies focusing on specific settings, such as upfront surgery for oropharyngeal p16-negative carcinoma, salvage surgery after radiotherapy failure, or salivary gland adenocarcinomas treatment, will help us to answer these questions.

## 5. Conclusions

In a large series of patients who underwent TORS and free flap microvascular reconstruction, we demonstrated technical feasibility in complex situations. Combined indications comprised large pharyngeal resections, the intra-operative exposition of critical structures, and previous head and neck radiotherapy. Peroperative feasibility was very satisfactory, although associated with a renewed learning curve. Postoperative clinical and functional outcomes were satisfactory in a population of high-burden patients at high risk of acute and chronic complications. TORS and free flap reconstruction have incorporated standard-of-care procedures at our institution.

## Figures and Tables

**Table 1:** Epidemiological and clinical characteristics of the first 50 patients who underwent transoral robotic surgery and a free flap reconstruction at our institution.

Characteristics		No. of Patients (%)
Age	Mean (range)	61.6 (29–80)
Gender	Male/Female	40 (80%)/10 (20%)
Charlson Comorbidity Index	Mean (min–max)	4.66 (3–10)
First head and neck localization		17 (34%)
Second primary cancer in the irradiated field		14 (28%)
Salvage surgery for radiotherapy failure		19 (38%)
Chronic tracheotomy/stomy for sequelae of a previous cancer		4 (8%)
Primary localizations	Tongue base	22 (44%)
	Tonsillar fossa	14 (28%)
	Pharyngeal wall	11 (22%)
	Soft palate	3 (6%)
Histologies	P16-negative SCC	39 (78%)
	P16-positive SCC	4 (8%)
	Salivary gland carcinoma	6 (12%)
	Synovial sarcoma	1 (2%)
Clinical T-classification	T1	3 (6%)
	T2	26 (52%)
	T3	10 (20%)
	T4	11 (22%)

**Table 2:** Surgical characteristics of the first 50 patients who underwent transoral robotic surgery and a free flap reconstruction at our institution.

Surgery Data		No. of Patients (%)
Previous tracheostomy or chronic tracheotomy		4 (8%)
Tracheotomy for TORS		46 (92%)
Mean length of surgery (range)	Minutes	574 min (293–820)
Interoperative conversion to an open approach		2 (4%)
Free flap	Thin anterolateral thigh flap	12 (24%)
	Standard anterolateral thigh flap	17 (34%)
	Radial forearm flap	10 (20%)
	Latissimus dorsi flap	2 (4%)
	Thoracodorsal artery perforator flap	4 (8%)
	Medial sural artery perforator flap	4 (8%)
	Superficial circumflex iliac artery perforator flap	1 (2%)
Median size of the defect measured transorally (range)	Centimeters	6 × 9 cm (5 × 6–12 × 13)
Mean greatest tumor dimension at pathological examination (range)	Millimeters	29.6 mm (12–70)

**Table 3:** Postoperative complication categories according to the stratification between surgery for first head and neck localization, surgery for second localization after previous radiotherapy, and salvage surgery for chemoradiotherapy failure.

Complication Categories		Number of Patients (%)	
	First Head and Neck Localization	Second Localization after Previous Radiotherapy	Salvage Surgery for Radiochemotherapy Failure
Total number of patients	17	14	19
Hemorragic complications	0	0	2 (10.5%)
Infectious local or general complication	5 (29.4%)	2 (14.3%)	4 (21.1%)
Healing troubles	5 (29.4%)	1 (7.1%)	2 (10.5%)
Reoperation for local complication	5 (29.4%)	1 (7.1%)	3 (15.8%)
Postoperative death	0	0	2 (10.5%)

## Data Availability

Data are not available.
